# Comparative *In Vitro* Study of Various α_2_-Adrenoreceptor Agonist Drugs for Ticagrelor Reversal

**DOI:** 10.3390/jcm9030809

**Published:** 2020-03-16

**Authors:** Guillaume Porta Bonete, Anne Godier, Pascale Gaussem, Tiphaine Belleville-Rolland, Alexandre Leuci, Sonia Poirault-Chassac, Christilla Bachelot-Loza, Anne-Céline Martin

**Affiliations:** 1 Université de Paris, Innovations Thérapeutiques en Hémostase, INSERM 1140, 75006 Paris, France; guillaume.portabonete@gmail.com (G.P.B.); anne.godier@aphp.fr (A.G.); pascale.gaussem@aphp.fr (P.G.); tiphainebelleville@gmail.com (T.B.-R.); aleuci2@gmail.com (A.L.); sonia.poirault-chassac@inserm.fr (S.P.-C.); christilla.bachelot-loza@parisdescartes.fr (C.B.-L.); 2 AP-HP, Service d’Anesthésie-Réanimation, Hôpital Européen Georges Pompidou, 75015 Paris, France; 3 AP-HP, Service d’Hématologie Biologique, Hôpital Européen Georges Pompidou, 75015 Paris, France; 4 AP-HP, Service de Cardiologie, Hôpital Européen Georges Pompidou, 75015 Paris, France

**Keywords:** antiplatelet agent, ticagrelor, reversal, α_2_-agonist

## Abstract

Ticagrelor, an antiplatelet adenosine diphosphate (ADP)-P2Y_12_ receptor antagonist, increases the risk of bleeding. Its management is challenging because platelet transfusion is ineffective and no specific antidote is currently available. Epinephrine, a vasopressor catecholamine prescribed during shock, restores platelet functions inhibited by ticagrelor through stimulation of α_2A_-adrenoreceptors. It subsequently inhibits cyclic adenosine monophosphate (cAMP) pathway and PI3K signaling. However, since epinephrine may expose a patient to deleterious hemodynamic effects, we hypothesized that other α_2_-adrenoreceptor agonist drugs used in clinical practice with fewer side effects could reverse the antiplatelet effects of ticagrelor. We compared *in vitro* the efficacy of clonidine, dexmedetomidine, brimonidine, and norepinephrine with epinephrine to restore ADP- and PAR-1-AP-induced washed platelet aggregation inhibited by ticagrelor, as well as resulting platelet cAMP levels. In ticagrelor-free samples, none of the α_2_-adrenoreceptor agonists induced aggregation by itself but all of them potentiated ADP-induced aggregation. Compared with epinephrine, norepinephrine, and brimonidine partially restored ADP- and fully restored PAR-1-AP-induced aggregation inhibited by ticagrelor while clonidine and dexmedetomidine were ineffective. Indeed, this lack of effect resulted from a lower decrease in cAMP concentration elicited by these partial α_2_-adrenoreceptor agonists, clonidine, and dexmedetomidine, compared with full α_2_-agonists. Our results support the development of specific full and systemic α_2_-adrenoreceptor agonists for ticagrelor reversal.

## 1. Introduction

Ticagrelor is a direct-acting, selective, and reversible antagonist of the platelet adenosine diphosphate (ADP) P2Y_12_ receptor that is recommended for the treatment of acute coronary syndromes [1]. Like any antithrombotic drug, ticagrelor exposes to bleeding complications, management of which is challenging. Indeed, ticagrelor is not dialysable and the circulating concentrations of ticagrelor and its active metabolite make platelet transfusion totally inefficient within at least 24 h after intake. Moreover, hemostatic agents including recombinant activated factor VII, tranexamic acid, and desmopressin are unlikely to reverse ticagrelor antiplatelet effects [2,3,4,5,6,7], and no specific antidote is currently available [8,9]. We recently showed that epinephrine, a vasopressor agent used to treat shock, is able *in vitro* to restore platelet functions inhibited by ticagrelor. Epinephrine acts through stimulation of α_2A_-adrenoreceptors coupled with G_z_-protein on the platelet membrane surface. The α subunit of G_z_-protein binds to adenylate cyclase and inhibits the synthesis of the second messenger cyclic adenosine monophosphate (cAMP), which plays a central role in platelet inhibition, while the dissociated βγ subunit activates phosphoinositide 3-kinase (PI3K) pathway [10,11]. Therefore, α_2A_-adrenoreceptor stimulation participates in platelet activation. In combination with ADP stimulating the P2Y_1_ receptor signaling and subsequent Ca^2+^ mobilization, epinephrine induces aggregation of ticagrelor-treated platelets through inhibition of the cAMP pathway and activation of the PI3K pathway. However, epinephrine is a catecholamine and cumulates α_1_-, α_2_-, β_1_-, and β_2_- adrenoreceptor agonist effects that induce hemodynamic changes including tachycardia, heart rhythm disorders, peripheral arterial vasoconstriction, and lactic acidosis, which are potentially deleterious in the context of acute bleeding. We therefore hypothesized that other α_2_-adrenoreceptor agonist drugs used in clinical practice in various settings with fewer side effects and less hemodynamic impact could also have platelet-activating properties and be of interest to reverse the antiplatelet effects of ticagrelor.

We selected 4 α_2_-adrenoreceptor agonists. Norepinephrine is a potent vasopressor acting through α_1_-adrenergic receptors recommended during hemorrhagic and septic shock. It also exerts α_2_- and β-agonist effects. Clonidine and dexmedetomidine are partial α_2_-adrenoreceptor agonists able to bind imidazoline receptors, dexmedetomidine being 200 times more powerful than clonidine. They exert broad pharmacological effects, including sedation, analgesia, anxiolysis, and sympathetic tone inhibition and are increasingly used in anesthesiology. Finally, brimonidine is a full imidazoline α_2_-adrenoreceptor agonist that is mainly used topically to reduce intraocular pressure in glaucoma or to treat erythema of rosacea. It also has hemodynamic properties on the heart and peripheral circulation [12].

We performed an *in vitro* study to compare the efficacy of these 4 α_2_-adrenergic receptor agonists with epinephrine in restoring platelet aggregation in the presence of ticagrelor.

## 2. Materials and Methods

Blood samples were obtained from the French Blood Bank Institute (Etablissement Français du Sang, Paris, France, convention ref. C CPSL UNT n°12/EFS/038) from healthy volunteers after obtaining written informed consent. Blood samples were collected in acid citrate dextrose (ACD) tubes (BD Vacutainer, citric acid 5.7 mM, trisodium citrate 11.2 mM, and dextrose 20 mM, final concentrations).

### 2.1. Isolation and Preparation of Washed Platelet Suspension

Experiments were performed on washed platelets in order to investigate the direct effects of α_2_-adrenoreceptor agonists on platelets, independently of the plasma proteins, and the anticoagulant used for blood sampling. The washed platelet suspension was prepared as previously described [11]: briefly, blood samples collected in ACD tubes were mixed with wash buffer (citric acid 36 mM, glucose 5 mM, KCl 5 mM, CaCl_2_ 2 mM, MgCl_2_ 1 mM, and NaCl 103 mM; pH = 6.5) containing 0.2 µM prostaglandin E1 (PGE1, Sigma-Aldrich, St. Louis, MO, USA) and 0.03 IU/mL apyrase (Agro-Bio, La Ferté-Saint-Aubin, France) to avoid platelet activation during preparation. Platelet-rich plasma was obtained after the first spin (216 g for 11 min at room temperature). Platelets were washed twice with wash buffer, containing PGE1 and apyrase, and centrifuged (1200× *g* for 11 min). The last pellet was resuspended in suspension buffer (Hepes 10 mM, NaCl 140 mM, KCl 3 mM, NaHCO_3_ 5 mM, MgCl_2_ 0.5 mM, and glucose 10 mM; pH = 7.35) to a final concentration of 3 × 10^8^ platelets/mL (=300 G/L, within the physiological range for platelet count). Apyrase (0.03 IU/mL) was added to prevent ADP receptor desensitization; then, CaCl_2_ 2 mM was added [13].

### 2.2. Agonists and Inhibitors

Ticagrelor was extracted from tablets (Brilique^®^ 90 mg, AstraZeneca, Mölndal, Sweden) as previously described [11]. Briefly, tablets were dispersed in 10 mM HCl and the water-soluble excipient extracted by centrifugation (5 min, 5000× *g*, 20 °C). The pellet was washed 3 times with distilled water followed by centrifugation (5 min, 5000× *g*, 20 °C). The pellet was resuspended in distilled water, snap-frozen in liquid nitrogen, and then lyophilized. Ticagrelor was extracted from the lyophilized material by incubation in pure DMSO. Insoluble material in the ticagrelor-saturated DMSO was removed by two centrifugations (5 min, 5000× *g*, 20 °C). The concentration of ticagrelor-saturated DMSO solution was approximately 100 mg/mL, and was diluted 1/10 in pure DMSO prior storage at −80 °C. Further dilutions were performed in suspension buffer containing 1% DMSO. The final DMSO concentration in all experiments was 0.001%. The final concentration of ticagrelor was assessed by reference to ticagrelor raw material provided by AstraZeneca (Mölndal, Sweden).

Washed platelets were incubated with ticagrelor for 10 min at 37 °C prior to use. A final concentration of 100 nM ticagrelor was selected from a dose-ranging study since it inhibited at least 80% of platelet aggregation [11]. ADP (Roche Molecular Biochemicals, Meylan, France) diluted in suspension buffer was used at either 5 µM final concentration, selected to induce less than 50% platelet aggregation in all samples, in order to capture potential α_2_-adrenoreceptor agonist synergism, or 10 µM final concentration, to maximize potential restoration in the presence of inhibitor. Since ADP acts as a second agonist released from the dense granules in response to strong platelet stimulation, we repeated the experiments using proteinase-activated receptor-1 activating peptide at 2 µM final concentration (PAR-1-AP, Bachem, Basel, Switzerland) as a primary agonist. Here, the aim was to use PAR-1-AP at a concentration sufficient to induce ADP release from dense granules and to assess subsequent ADP-dependent aggregation, not to induce maximal aggregation, independent of ADP, as observed at higher PAR-1-AP concentrations.

Epinephrine (Aguettant, Lyon Gerland, France) was diluted in suspension buffer and used at a final concentration of 10 µM which was selected from a dose-ranging study as the lowest concentration increasing ADP-induced platelet aggregation to a similar level to 10 µM PAR-1-AP, a surrogate for maximal activation and aggregation. Norepinephrine (Sigma-Aldrich, St. Louis, MO, USA), clonidine (Boehringer Ingelheim, Paris, France), dexmedetomidine (Baxter SA, Guyancourt, France), and brimonidine (Sigma-Aldrich, St. Louis, MO, USA) were diluted in suspension buffer and used at a final concentration of 10 µM (except dexmedetomidine, which cannot be dissolved at a higher concentration than 8 µM). For clonidine, dexmedetomidine, and brimonidine, final concentrations were selected according to the available data in vitro and dose-ranging studies.

### 2.3. Platelet Aggregation

Platelet aggregation was measured using light transmission aggregometry (PAP-8 platelet aggregometer, Biodata Corporation, Horsham, PA, USA): washed platelets (290 µL; 3 × 10^8^/mL) pre-treated with ticagrelor or vehicle were incubated for 2 min at 37 °C under stirring in the presence of fibrinogen (300 µg/mL, Hyphen Biomed, Neuville-sur-Oise, France). Aggregation was induced by adding the agonist (10 µM ADP or a combination of 10 µM ADP and 10 µM epinephrine, 10 µM norepinephrine, 10 µM clonidine, 8 µM dexmedetomidine, or 10 µM brimonidine), was recorded for 6 min, and the results were expressed as a percentage of the maximal aggregation. Experiments were repeated using 2 µM PAR-1-AP as the primary agonist.

### 2.4. Measurement of Intracellular cAMP

Intracellular cAMP concentration was measured using the cAMP kit (Cisbio Bioassays, Codolet, France) based on an immuno-enzymatic method, according to the manufacturer’s protocol. Briefly, 40 μL washed platelets (5 × 10^8^ platelets/mL) pre-treated with ticagrelor or vehicle (resuspension buffer/DMSO) were incubated with 5 μL PGE1 (final concentration 1 μM) allowing cAMP production in experimental conditions. After 30 s, 5 μL of agonists (ADP or a combination of ADP and α_2_-adrenoreceptor agonists) were added, and the reaction was stopped 1 min and 3 min later using 50 μL lysis buffer containing the phosphodiesterase pan-inhibitor 3-isobutyl-1-methylxanthine (final concentration 2 mM). The samples were then centrifuged at 4 °C for 1 min at 13,200 rpm and the supernatant was stored at −20 °C, and subsequently thawed immediately prior testing. The results were expressed in picomoles adjusted to 10^9^ platelets (pmol/10^9^ platelets).

### 2.5. Statistical Analysis

Quantitative data were expressed as medians (minimum–maximum) or box-and-whisker plots showing median, interquartile range, minimum, and maximum values. Statistical analysis was performed using Prism v6.0 (GraphPad software, San Diego, CA, USA). Nonparametric tests were used for comparisons. Variables were compared using the Friedman test, followed by the Wilcoxon test (paired samples) or the Mann–Whitney (independent samples). All statistical tests were two-sided and *p* < 0.05 was considered statistically significant.

## 3. Results

### 3.1. All α_2_-Adrenoreceptor Agonists Potentiate ADP-Induced Platelet Aggregation in the Absence of Ticagrelor

**Figure 1:**
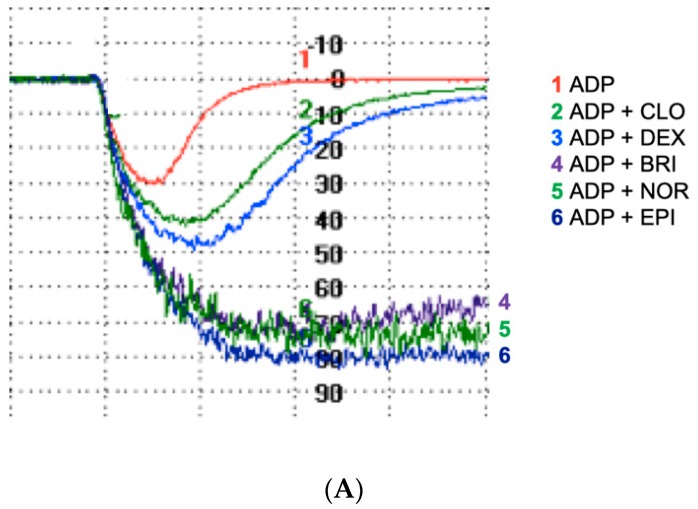

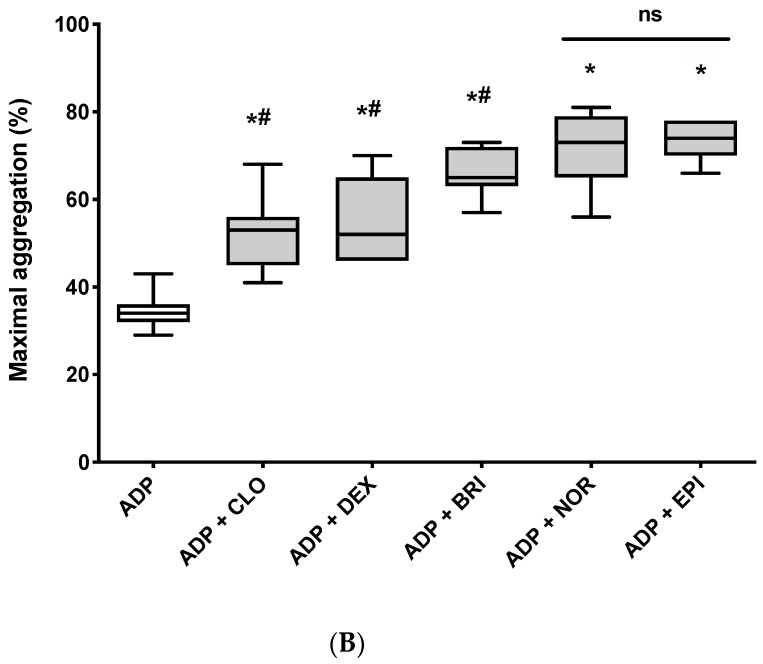
All α2-adrenoreceptor agonists potentiate adenosine diphosphate (ADP)-induced platelet aggregation in the absence of ticagrelor. Washed platelet aggregation on stimulation with different agonists (5 µM ADP alone or in combination with clonidine (CLO, 10 µM), dexmedetomidine (DEX, 8 µM), brimonidine (BRI, 10 µM), norepinephrine (NOR, 10 µM), or epinephrine (EPI, 10 µM)). (**A**) Typical time-course of platelet aggregation using light transmission aggregometry. (**B**) Comparison of percentage of the maximal aggregation (*n* = 7). Boxes represent the interquartile range and the median value, whiskers and the minimum and maximum values observed. All samples were compared with ADP alone (* *p* < 0.01 vs. ADP alone), and with the combination of ADP and EPI (# *p* < 0.05 vs. ADP + EPI).

As expected, in the presence of fibrinogen, the weak agonist ADP induced reversible platelet aggregation while none of the α_2_-adrenoreceptor agonists induced significant aggregation when used alone (data not shown). Combined with ADP, each α_2_-adrenoreceptor agonist induced a significantly stronger aggregation than ADP alone (Figure 1A,B). Norepinephrine potentiated ADP-induced aggregation (73% (56–81) vs. 34% (29–43), *p* < 0.001) that achieved the level induced by epinephrine (74% (66–78) vs. 73% (56–81), *p* = 0.97), and displayed an irreversible aggregation profile. Brimonidine, clonidine, and dexmedetomidine also potentiated ADP-induced aggregation (65% (57–81), 53% (41–68), and 52% (46–70) vs. 34% (29–43), *p* < 0.001, *p* < 0.01, *p* < 0.001, respectively). However, their effects were significantly lower than epinephrine (*p* < 0.001 for the comparison of each α_2_-adrenoreceptor agonist with epinephrine). Whereas clonidine and dexmedetomidine combined with ADP induced a reversible aggregation like ADP alone, brimonidine induced irreversible aggregation like epinephrine and norepinephrine (Figure 1A).

### 3.2. Norepinephrine and Brimonidine Partially Restore ADP- and PAR-1-AP-Induced Platelet Aggregation Inhibited by Ticagrelor, While Clonidine and Dexmedetomidine Are Ineffective

**Figure 2:**
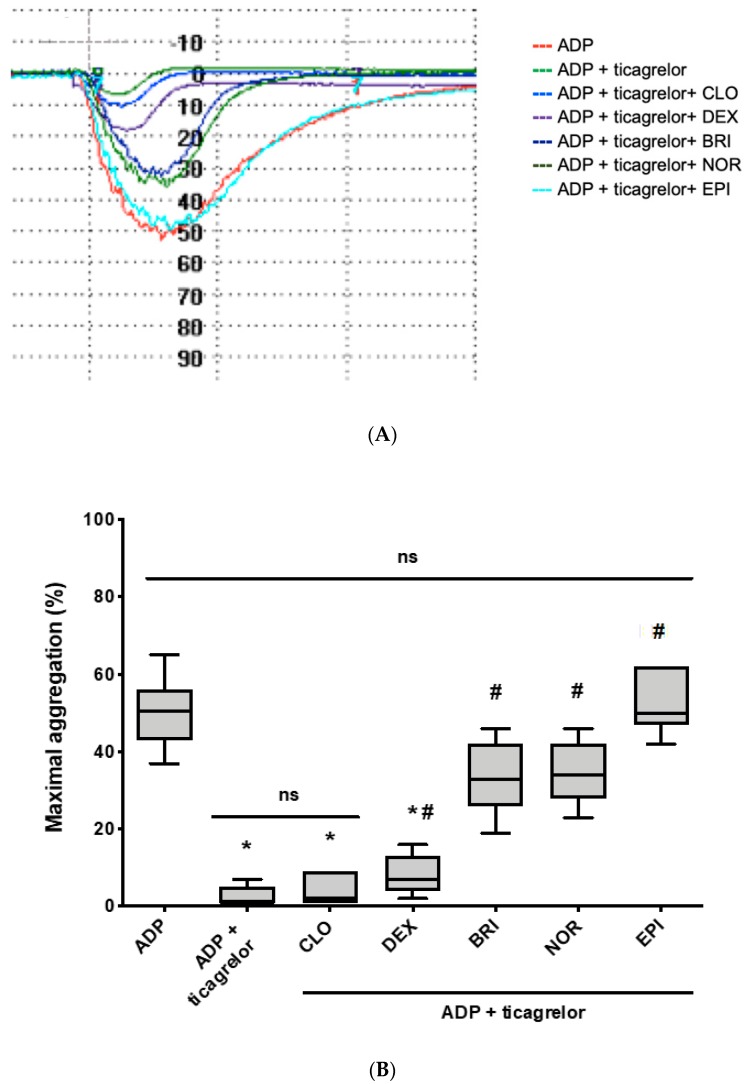
Norepinephrine and brimonidine partially restore ADP-induced platelet aggregation inhibited by ticagrelor, while clonidine and dexmedetomidine are ineffective. Washed platelet aggregation on stimulation with different agonists (10 µM ADP alone or in combination with clonidine (CLO, 10 µM), dexmedetomidine (DEX, 8 µM), brimonidine (BRI, 10 µM), norepinephrine (NOR, 10 µM), or epinephrine (EPI, 10 µM)), in the absence or presence of ticagrelor. (**A**) Typical time-course of platelet aggregation using light transmission aggregometry. (**B**) Comparison of percentage of the maximal aggregation (*n* = 7). Boxes represent the interquartile range and the median value, whiskers and the minimum and maximum values observed. All samples were compared with ADP alone (* *p* < 0.01 vs. ADP alone) or ADP in the presence of ticagrelor (# *p* < 0.05 vs. ADP + ticagrelor).

As expected, ticagrelor-inhibited ADP-induced platelet aggregation (1% (1–7) vs. 51% (37–65), *p* < 0.05), which was fully restored by epinephrine (50% (42–62) vs. 51% (37–65), *p* = 0.97) (Figure 2A,B). Norepinephrine and brimonidine significantly increased ADP-induced platelet aggregation inhibited by ticagrelor (34% (23–46) and 33% (19–46) vs. 1% (1–7), *p* < 0.001 for both); however, restoration was partial (*p* < 0.001 for both, compared to ADP without ticagrelor) and less efficient than that induced by epinephrine (*p* < 0.01 for both). On the contrary, clonidine and dexmedetomidine failed to correct ADP-induced platelet aggregation inhibited by ticagrelor although a faint increase was observed (2% (1–9), *p* = 0.36 and 4% (2–16), *p* = 0.04, respectively).

**Figure 3:**
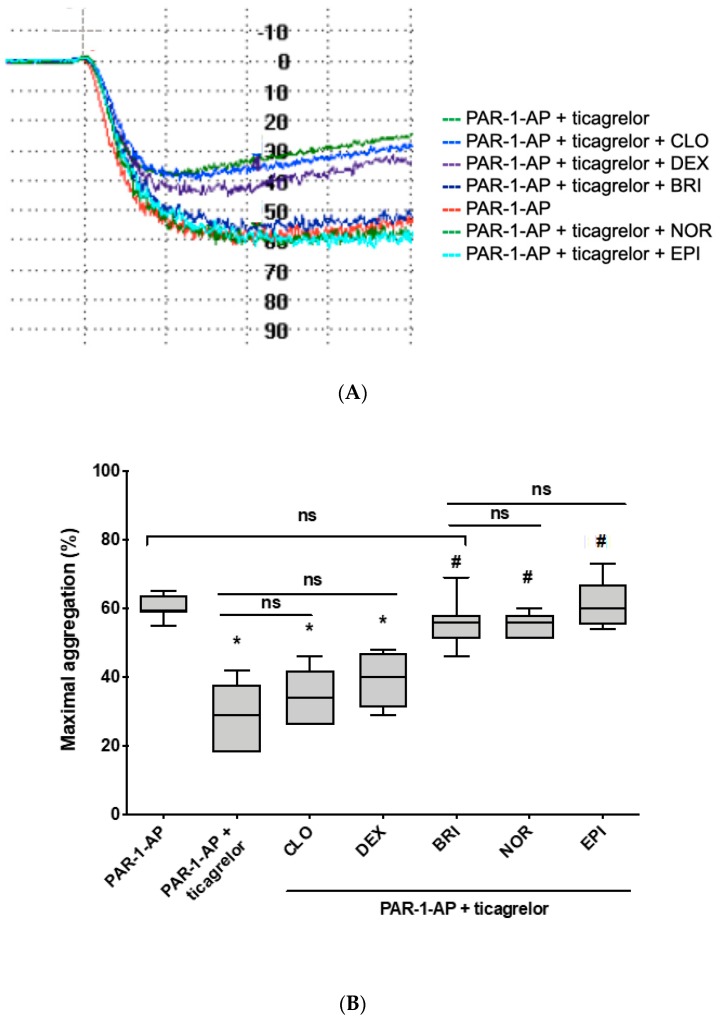
Norepinephrine and brimonidine restore PAR-1-AP-induced platelet aggregation inhibited by ticagrelor, while clonidine and dexmedetomidine are ineffective. Washed platelet aggregation on stimulation with different agonists (2 µM PAR-1-AP alone or in combination with clonidine (CLO, 10 µM), dexmedetomidine (DEX, 8 µM), brimonidine (BRI, 10 µM), norepinephrine (NOR, 10 µM), or epinephrine (EPI, 10 µM)), in the presence of ticagrelor. (**A**) Typical time-course of platelet aggregation using light transmission aggregometry. (**B**) Comparison of percentage of the maximal aggregation (*n* = 7). Boxes represent the interquartile range and the median value, whiskers and the minimum and maximum values observed. All samples were compared with PAR-1-AP alone (* *p* < 0.01 vs. PAR-1-AP alone) or PAR-1-AP in the presence of ticagrelor (# *p* < 0.05 vs. PAR-1-AP + ticagrelor).

In a more physiological model, when PAR-1-AP was used as a primary agonist, ticagrelor partially inhibited PAR-1-AP-induced platelet aggregation (29% (18–42) vs. 59% (55–65), *p* < 0.001), which was fully restored by adding epinephrine (60% (54–73) vs. 59% (55–65), *p* = 0.88). Norepinephrine and brimonidine succeeded in restoring PAR1-ap-induced platelet aggregation inhibited by ticagrelor, at the same level as epinephrine (respectively, 56% (51–60) vs. 60% (54–73), *p* = 0.09, and 56% (46–69) vs. 60% (54–73), *p* = 0.16) (Figure 3A,B). On the contrary and similar to what was observed on ADP stimulation, clonidine and dexmedetomidine failed to restore aggregation. Indeed, no significant increase was observed compared with PAR-1-AP-induced platelet aggregation inhibited by ticagrelor (34% (26–46) and 40% (29–48) vs. 29% (18–42), *p* = 0.31 and *p* = 0.07, respectively).

### 3.3. Norepinephrine and Brimonidine Fully Inhibit PGE1-Stimulated cAMP Production Whereas Clonidine and Dexmedetomidine Induce Only Weak Inhibition

**Figure 4:**
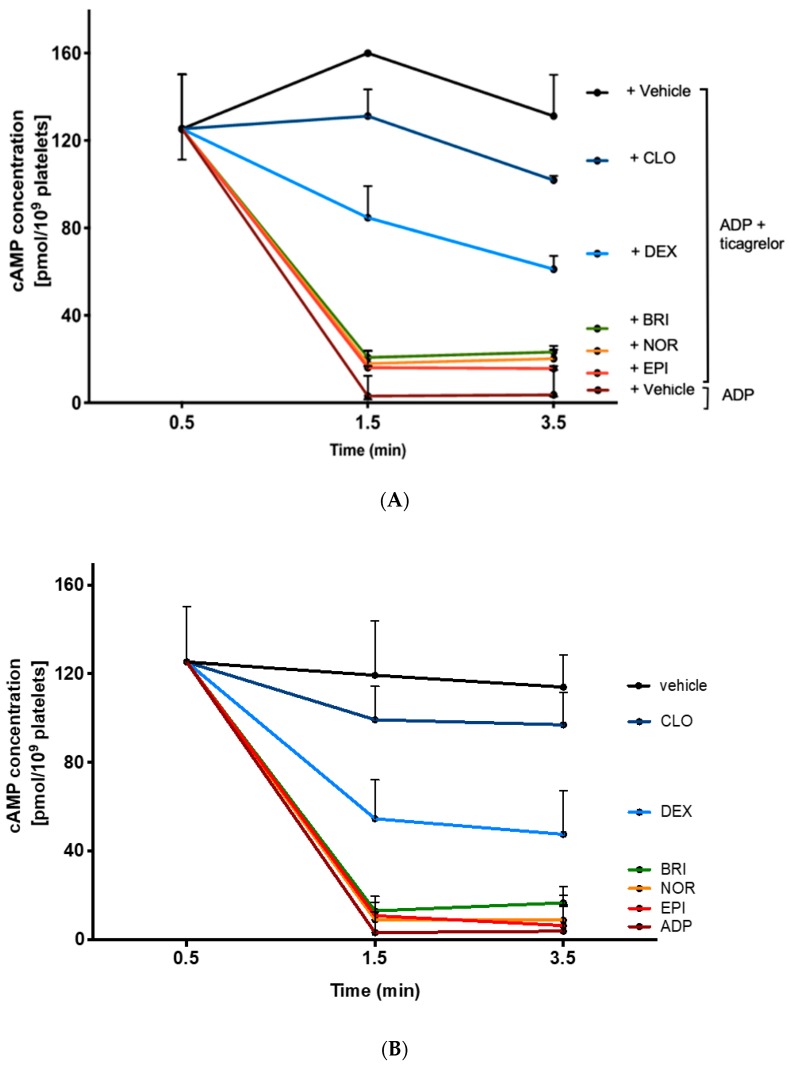
Norepinephrine and brimonidine fully inhibit PGE1-stimulated cAMP production whereas clonidine and dexmedetomidine induce only weak inhibition. Comparison of cAMP level of washed platelet pre-incubated with PGE1 for 0.5 min and then stimulated with (**A**) ADP alone, or a combination of ADP and clonidine (CLO), dexmedetomidine (DEX), brimonidine (BRI), norepinephrine (NOR), or epinephrine (EPI), in the presence of ticagrelor and with (**B**) ADP or α_2_-adrenoreceptor agonists, in the absence of ticagrelor. cAMP assays were performed on samples taken before (time 0.5 min), or 1 min and 3 min (time 1.5 min and 3.5 min) after the addition of the agonists or vehicle. Results are expressed in the median with ranges (*n* = 3).

To further explore the differences between various α_2_-adrenoreceptor agonists in restoring platelet aggregation inhibited by ticagrelor, we assessed the level of cAMP in platelets sensitized with PGE1 (cAMP level < 20 pmol/10^9^ platelets before PGE1 sensitization) and stimulated under various conditions. As expected, ADP decreased cAMP level compared with control (4 (3–15) vs. 114 (81–128) pmol/10^9^ platelets) and ticagrelor-inhibited ADP-induced suppression of intracellular cAMP (131 (97–150) vs. 4 (3–15) pmol/10^9^ platelets, Figure 4A). Combining ADP and either epinephrine, norepinephrine, or brimonidine in the presence of ticagrelor fully restored suppression of intracellular cAMP production, achieving the same level as obtained with ADP alone in the absence of ticagrelor (16 (9–17), 20 (15–26), or 23 (17–24) pmol/10^9^ platelets, respectively). Adding dexmedetomidine induced a moderate reduction in cAMP concentration compared with the combination of ADP and ticagrelor (61 (53–67) vs. 131 (97–150) pmol/10^9^ platelets) whereas clonidine had only a faint effect (102 (88–104) vs. 131 (97–150) pmol/10^9^ platelets). Figure 4B shows that α_2_-adrenoreceptor agonists decreased cAMP level regardless of the presence of ticagrelor or ADP. This result confirmed that α_2_-adrenoreceptor agonists inhibited activation of cAMP pathway, via α_2A_-adrenergic-coupled G_z_, independently of other signaling pathways, especially those induced by P2Y_12_ activation.

## 4. Discussion

Previous studies investigating the *in vitro* effects of α_2_-adrenoreceptor agonists on platelet functions mainly focused on epinephrine. In the present study, we aimed to compare the effects on platelet aggregation of four marketed α_2_-adrenoreceptor agonists compared with those of epinephrine. We confirmed that all α_2_-adrenoreceptor agonists potentiated ADP-induced platelet aggregation. More importantly, we provided evidence that norepinephrine or brimonidine in synergism with ADP restored platelet aggregation inhibited by ticagrelor with the same efficacy as epinephrine, whereas dexmedetomidine and clonidine failed to overcome the inhibitory effect of ticagrelor.

Several mechanistic hypotheses may be advanced to explain these discrepancies among α_2_-adrenoreceptor agonists. First, it is well known that cAMP is the main intracellular regulator of platelet activation and consecutively of platelet aggregation. Dexmedetomidine and clonidine may induce insufficient suppression of cAMP production to elicit platelet aggregation. Indeed, we observed that dexmedetomidine and clonidine had a weak effect on the intracellular cAMP level unlike epinephrine, norepinephrine, and brimonidine. This is in line with the pharmacological characteristics identifying full α_2_-adrenoreceptor agonists, including norepinephrine and brimonidine, and partial α_2_-adrenoreceptor agonists, including dexmedetomidine and clonidine. Kafka et al. demonstrated that clonidine was as potent as epinephrine and norepinephrine in binding to the receptor but was only 1/10 as potent in inhibiting PGE1-stimulated cAMP production [14]. Second, we previously showed that restoration of platelet aggregation by epinephrine is due to cAMP pathway inhibition and PI3K pathway activation, but simultaneously requires ADP-induced P2Y_1_-receptor-related signaling and Ca^2+^ mobilization [11]. We cannot exclude that dexmedetomidine and clonidine exert only partial activation of the PI3K pathway, thereby limiting subsequent signaling involved in sustained activation, granule secretion, and stable thrombus formation.

Finally, dexmedetomidine and clonidine are able to bind I_1_-imidazoline receptors, distinct from α_2_-adrenoreceptors on the platelet membrane surface [15,16]. Since I_1_-receptor stimulation induces guanylate cyclase activation and the subsequent increase in the intracellular cGMP level, dexmedetomidine and clonidine can also participate in platelet aggregation inhibition [17]. Consequently, dexmedetomidine and clonidine have both enhancing and suppressive effects on platelet functions through their opposite action on α_2_-adrenoceptors and on I_1_-imidazoline receptors, respectively [18]. In our experimental conditions, these two antagonist mechanisms resulted in potentiation of ADP-induced platelet aggregation insufficient to induce platelet aggregation in the presence of ticagrelor. We nevertheless noted that dexmedetomidine was more efficient than clonidine.

These *in vitro* findings translated into conflicting *in vivo* observations. Adam et al. showed that clonidine added *ex vivo* to samples from patients treated with dual antiplatelet therapy (aspirin and clopidogrel) did not affect ADP-induced platelet aggregation compared with control [19]. However, Janatmakan et al. recently reported in a randomized study conducted in 120 patients undergoing spinal surgery that clonidine or dexmedetomidine reduced intraoperative blood loss compared with the control group. However, none of the patients received antiplatelet agents [20]. In contrast, Mizrak et al. reported that dexmedetomidine increased bleeding in pediatric patients undergoing adenotonsillectomy [21].

Our study aimed to identify potential α_2_-adrenoreceptor agonists as efficient as epinephrine in restoring ticagrelor-inhibited platelet aggregation *in vitro*, but with fewer deleterious hemodynamic effects. So far studies performed with epinephrine are encouraging. Using a model of artery stenosis, Yao et al. and Samama et al. have reported in canines and pigs, respectively, that epinephrine infusion fully restored platelet thrombus formation abolished by clopidogrel [22,23] Singh et al. recently confirmed that even low clinically-relevant doses of epinephrine infusion (0.15 µg/kg/min, leading to epinephrine concentration 20 nM) slightly improved platelet aggregation and clot formation in healthy volunteers receiving ticagrelor [24]. However, it is likely that higher epinephrine doses would be required to translate biological surrogates into clinically-relevant effects on bleeding control, exposing patients to hypertension and bleeding enhancement, and serious cerebral and cardiovascular adverse effects [25]. Norepinephrine has not been assessed with the aim to restore hemostasis. However, norepinephrine is a catecholamine cumulating strong α_1_-, α_2_-, and β_-_ effects, and 10 times less potent than epinephrine suggesting a supratherapeutic dose to exert potential platelet effects, exposing patients to unacceptable hemodynamic effects. Finally, the more attractive candidate was brimonidine, a pure full α_2_-adrenoreceptor agonist. However, its restricted cutaneous or intraocular administration limits the scope of its use to restore platelet function inhibited by ticagrelor.

## 5. Study Limitations and Conclusions

Regarding limitations, we acknowledge that we assessed a way to improve platelet functions inhibited by ticagrelor rather than a specific antidote, which is in development but not marketed yet. The main weakness of our study lies in the *in vitro* design, which makes it difficult to transpose our findings to the clinical management of ticagrelor-induced bleeding. To go further, it would be interesting to assess the effects of α_2_-adrenoreceptor agonists on various assays using platelets from ticagrelor-treated patients.

Norepinephrine, recommended for hemorrhagic shock, may expose patients to side effects, especially in the absence of shock, and brimonidine is limited by its topical administration. Further research is required to assess the reversal effects of these drugs in the clinical setting and to support the development of pure full and systemic α_2_-adrenoreceptor agonists for ticagrelor reversal.
